# Role of AMPK signalling pathway during compensatory growth in pigs

**DOI:** 10.1186/s12864-018-5071-5

**Published:** 2018-09-17

**Authors:** Maria Ballester, Marcel Amills, Olga González-Rodríguez, Tainã F. Cardoso, Mariam Pascual, Rayner González-Prendes, Núria Panella-Riera, Isabel Díaz, Joan Tibau, Raquel Quintanilla

**Affiliations:** 1 Animal Breeding and Genetics Programme, Institute for Research and Technology in Food and Agriculture (IRTA), Torre Marimon, E08140 Caldes de Montbui, Spain; 2 Department of Animal Genetics, Center for Research in Agricultural Genomics (CSIC-IRTA-UAB-UB), Universitat Autònoma de Barcelona, E08193 Bellaterra, Spain; 3 Product Quality Programme, Institute for Research and Technology in Food and Agriculture (IRTA), Finca Camps i Armet, E17121 Monells, Spain; 4 Animal Breeding and Genetics Programme, Institute for Research and Technology in Food and Agriculture (IRTA), Finca Camps i Armet, E17121 Monells, Spain

**Keywords:** Pig, Feed restriction, Compensatory growth, Skeletal muscle, RNA-Seq, Autophagy, Energy homeostasis, AMPK

## Abstract

**Background:**

The molecular basis of compensatory growth in monogastric animals has not yet been fully explored. Herewith, in this study we aim to determine changes in the pig skeletal muscle transcriptome profile during compensatory growth following a feed restriction period. A RNA-Seq experiment was performed with a total of 24 females belonging to a Duroc commercial line. Half of the animals received either a restricted (RE) or ad libitum (AL) diet during the first fattening period (60–125 d of age). After that, all gilts were fed ad libitum for a further ~30 d until the age of ~155 d, when animals were slaughtered and samples of *gluteus medius* muscle were harvested to perform RNA-Seq analyses and intramuscular fat content determination.

**Results:**

During the period following food restriction, RE animals re-fed ad libitum displayed compensatory growth, showed better feed conversion rate and tended to deposit more subcutaneous fat than AL fed animals. Animals were slaughtered in the phase of accelerated growth, when RE animals had not completely compensated the performance of AL group, showing lower live and carcass weights. At intramuscular level, RE gilts showed a higher content of polyunsaturated fatty acids during the compensatory growth phase. The comparison of RE and AL expression profiles allowed the identification of 86 (ǀlog_2_Fold-Changeǀ > 1, p_adj_ < 0.05) differentially expressed (DE) genes. A functional categorization of these DE genes identified AMPK Signaling as the most significantly enriched canonical pathway. This kinase plays a key role in the maintenance of energy homeostasis as well as in the activation of autophagy. Among the DE genes identified as components of AMPK Signaling pathway, five out of six genes were downregulated in RE pigs.

**Conclusions:**

Animals re-fed after a restriction period exhibited a less oxidative metabolic profile and catabolic processes in muscle than animals fed ad libitum. The downregulation of autophagy observed in the skeletal muscle of pigs undergoing compensatory growth may constitute a mechanism to increase muscle mass thus ensuring an accelerated growth rate. These results reveal that the downregulation of AMPK Signaling plays an important role in compensatory growth in pigs.

**Electronic supplementary material:**

The online version of this article (10.1186/s12864-018-5071-5) contains supplementary material, which is available to authorized users.

## Background

In the pork industry, different feeding strategies have been applied to modify growth performance, carcass composition and meat quality traits (reviewed in [1, 2]). Among these strategies, the benefits of having a compensatory growth (CG) response induced by an energy and/or nutrient intake restriction period followed by subsequent ad libitum feeding have been explored in several studies [2–6]. There is, however, some controversy regarding the possible benefits of feed restriction on final performance. Despite initial decreases in growth rates and fat deposition, the CG phenomenon has been studied for several decades in pigs (reviewed in [7]). The magnitude of compensation (partial, complete or no CG) is affected by multiple factors such as the type, degree, timing and duration of growth restriction, body development at the beginning of the restriction period and genotype (reviewed in [7, 8]). As one main benefit, some studies pointed out an improvement in feed efficiency in animals under restriction [2], and in some cases the reduction of back fat could be considered as desirable.

Over the last decades, the amount and composition of fat in muscle have gained special interest in the food industry due to their profound effect on meat quality. Intramuscular fat content and composition directly impact the nutritional quality of food as well as its sensory attributes which play an integral role in the overall consumer acceptance, such as meat tenderness and flavour [9, 10]. The effect of feed restriction on muscle fat deposition and fatty acid profile should be investigated, especially in systems aimed at obtaining a product of differential quality. To this extent, Heyer and Lebret [11] reported that food restriction did not cause significant changes on intramuscular fat content, whereas Daza et al. [2] observed changes in the muscle fatty acid profile of Iberian pigs under food restriction.

Recently, Keogh et al. [12] have described changes in the bovine skeletal muscle profile of cattle under restriction and during the subsequent early CG using RNA-Seq. These studies indicated that the ß-oxidation of fatty acids, oxidative phosphorylation and the tricarboxylic acids cycle were upregulated in restricted cattle, but the direction of the expression changes reverses during the re-feeding and CG phase [12]. However, the molecular basis of CG in ruminants and monogastrics could be very different. Although different studies have described the physiological aspects associated with feed restriction and CG in several species [8, 13], there is a lack of literature analysing gene expression changes associated to CG in pigs. The objective of this study is to gain new insights into the biological and molecular mechanisms underlying CG and phenotypic changes induced by feed restriction in pigs.

## Methods

### Animals and experiment

An experimental device was set up for evaluating changes in the muscle transcriptome of pigs during early CG induced by feed restriction followed by subsequent ad libitum feeding. On the basis of previous observations of CG in conventional pig breeds or crossbreds (reviewed in Lebret [1]), the refeeding period after restriction was established in 30–35 d to ensure that animals were in the phase of accelerated growth. The experiment was carried out with 24 gilts from a commercial Duroc line which is devoted to produce high quality cured products. These 24 females were born in the same week (25th – 31st January 2015) in 12 different litters, i.e. 12 pairs of full sibs. After weaning at 3–4 weeks of age, female piglets were moved from the farm of origin to the IRTA (Institut de Recerca i Tecnologia Agroalimentàries)-Pig Experimental Farm in Monells (Girona, Spain). At their arrival, animals were housed in transition devices and fed ad libitum a standard transition diet until approximately 2 months of age (around 18 kg of live weight). Gilts were then transferred to the fattening pens, where they were housed individually and distributed in two dietary conditions for the first part of the fattening period: fed ad libitum (AL group) and fed under restriction (RE group). Each of the sibling pairs were divided into the two dietary treatments, so that the 12 gilts in one group were full sibs of the 12 gilts in the other. Gilts were housed individually and fed with the same standard grower feed (Additional file 1: Table S1). During the first part of fattening period, between 60 and 125 d of age, animals in RE group received a quantity of feed limited to 75–80% of their estimated needs based on weight, whereas animals from AL group were fed ad libitum. Subsequently, all animals were fed ad libitum for a period of 30–35 d, until ~5 months of age (161±0.5 d of age; 96.8 ± 1.8 kg of live weight), when they were slaughtered. Pigs were weighed individually at the beginning and every 2 weeks during the whole experiment (at the age of 8, 10, 12, 14, 16, 18, 20 and 22 weeks), plus the day before slaughtering. Additionally, back fat thickness (BFT) in vivo was measured with a PIGLOG 105 ultrasound equipment in the third (~30 kg of live weight) and successive controls (i.e. at 12, 14, 16, 18, 20 and 22 weeks of age approximately). Individual feed intake during the two periods of the trial (feed restriction and realimentation/CG periods) was also recorded for all RE and AL gilts. Individual growth and feed conversion ratio during each period were computed.

Animals were slaughtered at IRTA Experimental Slaughterhouse in Monells (Girona, Spain) in totally controlled conditions and in compliance with all welfare regulations. Before slaughter, all RE and AL sows were fasted for 12 h and stunned with high concentrations of CO_2_ before bleeding. All experimental procedures were approved by the Ethical Committee of IRTA.

### Phenotypic data

After slaughtering, carcass weight and AutoFOM2 measures of lean percentage, loin thickness and BFT between the third and fourth ribs were registered at the slaughterhouse. Moreover, samples of ~100 g of *gluteus medius* (GM) muscle were collected for laboratory analyses. Electric conductivity (EC), ultimate pH (pH_24_), and meat colour parameters (lightness L*, redness a* and yellowness b*) were determined 24-h after slaughtering following the methods described in Gonzalez-Prendes et al. [14]. Analyses of GM lipid components included the determination of percentage of intramuscular fat (IMF), cholesterol content and fatty acid composition in the C12 - C22 interval, as described in Canovas et al. [15]. Statistical differences between group means were assessed with a two-tailed t-test.

In order to perform RNA-Seq analysis, additional GM muscle samples were collected immediately after slaughter and submerged in RNAlater (Sigma, Spain) before storage at − 80 °C, according to the protocols recommended by the manufacturer.

### RNA extraction, library construction and sequencing

The GM samples were individually ground with a mortar and a pestle to homogenization on liquid nitrogen. The RNA of the samples was extracted using the Ambion RiboPure (Thermo Fisher Scientific). Total RNA was quantified in a Nanodrop ND-1000 spectrophotometer and RNA purity and integrity was checked by using a Bioanalyzer-2100 equipment (Agilent Technologies, INC., Santa Clara, CA). Libraries were prepared using the TruSeq RNA Sample Preparation Kit (Ilumina Inc., CA). Pools of three libraries per line with barcoding were paired-end sequenced (2 × 75 bp), by using the TruSeq SBS Kit v3-HS (Illumina Inc., CA), in a HiSeq 2000 platform (Illumina Inc., CA). All sequencing tasks were carried out in the Centro Nacional de Análisis Genómico (Barcelona, Spain).

### Mapping, annotation and differential expression analysis

The quality of the raw sequenced reads in the FASTQ files was analysed with the FASTQC software (Babraham Bioinformatics, http://www.bioinformatics.babraham.ac.uk/projects/fastqc/). Reads were mapped to the reference pig genome Sscrofa10.2 and the annotation database Ensembl Genes 86 (http://www.ensembl.org/info/data/ftp/index.html) by using STAR v. 2.5.2a [16]. Mapping quality evaluation and descriptive statistics were assessed with Qualimap v. 2.2. [17]. The number of reads mapping to each gene were obtained with the HTSeq-count tool included in the HTSeq python library [18] by using the same GTF file of the alignment step. The R package DESeq2 [19] was used to identify differentially expressed (DE) genes based on the RNA-seq data. Genes with a Fold Change (FC) above 2 (i.e. ǀlog_2_FCǀ > 1) and significant *P*-value after correcting for multiple testing (*P*_adj_-value < 0.05) were classified as DE. Gene ontologies (GO), metabolic pathways, and biological functions significantly (*p*_adj_ < 0.05) enriched in the set of DE genes were determined using the ClueGO v. 2.3.5 plug-in of Cytoscape v. 3.2.1 [20] and the Core Analysis function included in the Ingenuity Pathway Analysis software (IPA; Ingenuity Systems). For functional analyses, orthologous human gene names were retrieved from the Ensembl Genes 89 Database using the Biomart software [21].

## Results

### Phenotypic differences

**Fig. 1:**
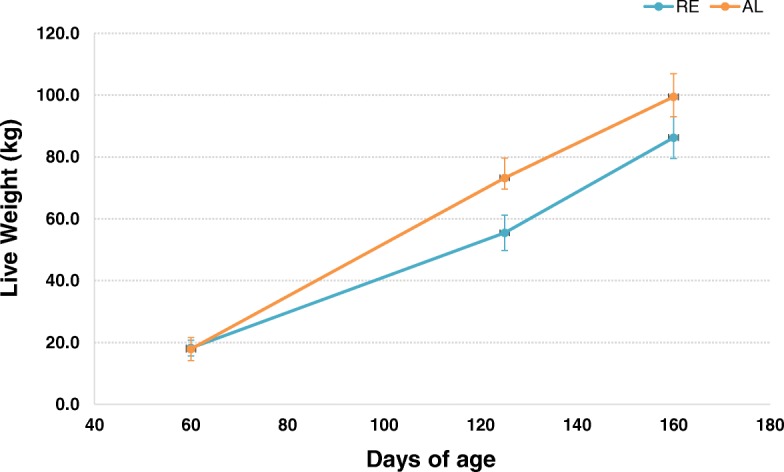
Evolution of live weight of animals subjected to feed restriction from 70 to 125 d of age (RE animals) and fed ad libitum (AL animals) across the controlled fattening period till sacrifice (155 d of age)

Phenotypic means of RE and AL groups for production and meat quality traits are presented in Table 1. Our results show that RE animals had significantly lower growth rates than the AL ones during the restriction period but they experienced compensatory growth when they were fed ad libitum, thus surpassing daily gains obtained in AL group (1.14 kg in RE vs 0.97 kg in AL; *P*-value < 0.005). Growth curves for both RE and AL animals are shown in Fig. 1. After ~35 d of re-feeding, the “compensatory index value” in our animals was 26% (calculated as reviewed in [13]). In addition, no significant differences in feed intake were observed between groups when both RE and AL gilts were fed ad libitum. Such observation is explained by the better feed conversion rate of RE gilts during CG (2.53 vs 2.93 g intake / g gain in RE and AL groups respectively; *P*-value < 0.001).

**Table 1:** Mean phenotypic values for growth, fatness, carcass and intramuscular fat traits of the two groups of pig females analysed, fed ad libitum (AL group) and fed a restricted diet (RE group), plus the significance of dietary treatment effect in a one way ANOVA

	AL	RE	*P*-value^a^
GROWTH from 70 to 125 d of age *(restriction period)*			
Average daily gain (kg)	0.88	0.55	<.0001 ***
Daily feed intake (kg)	2.05	1.26	<.0001 ***
Food conversion ratio (kg intake / kg gain)	2.33	2.27	0.2863 n.s.
Back fat gain from 85 to 125 d (mm)	5.25	2.75	<.0001 ***
GROWTH from 126 to 155 d of age *(re-feeding period)*			
Average daily gain (kg)	0.97	1.14	0.0046 **
Daily feed intake (kg)	2.84	2.88	0.8060 n.s.
Food conversion ratio (kg intake / kg gain)	2.93	2.53	0.0005 ***
Back fat gain (mm)	5.00	6.21	0.0569 +
CARCASS TRAITS (~ 161 d of age)			
Live weight before slaughter (kg)	101.10	89.10	<.0001 ***
Killing out (%)	80.03	79.03	0.0640 +
Chilling losses (%)	3.01	3.59	0.0015 **
Back fat thickness between 3rd and 4th ribs (Fat-O-Meater II) (mm)	25.52	22.28	0.0243 *
Loin thickness between 3rd and 4th ribs (Fat-O-Meater II) (mm)	44.13	40.30	0.0239 *
Lean meat percentage (FOM 2)	50.16	52.33	0.0851 +
INTRAMUSCULAR FAT TRAITS (*gluteus medius*)			
Intramuscular fat content (%)	3.733	3.120	0.0542 +
Cholesterol content (dg/kg)	58.082	58.193	0.9695 n.s.
Myristic acid (C14:0) content (%)	1.348	1.291	0.3641 n.s.
Palmitic acid (C16:0) content (%)	23.844	23.326	0.1258 n.s.
Palmitoleic acid (C16:1n7) content (%)	3.003	2.662	0.0164 *
Stearic acid (C18:0) content (%)	13.713	13.926	0.6280 n.s.
Oleic acid (C18:1n9) content (%)	40.100	38.454	0.0806 +
Cis-vaccenic acid (C18:1n7) content (%)	3.960	3.853	0.1893 n.s.
Linoleic acid (C18:2n6) content (%)	8.531	10.185	0.0110 *
Arachidonic acid (C20:4n6) content (%)	1.440	1.874	0.0630 +
Saturated Fatty Acids content (SFA) (%)	39.403	38.991	0.5857 n.s.
Monounsaturated fatty acids content (MUFA) (%)	49.249	47.156	0.0529 +
Polyunsaturated fatty acids content (PUFA) (%)	11.347	13.818	0.0149 *
Ratio PUFA/SFA	0.288	0.356	0.0158 *
Omega 6 (ω6) content	8.851	10.832	0.0089 **
Omega 3 (ω3) content	0.933	1.270	0.1762 n.s.
Ratio ω6/ ω3	9.935	9.816	0.9016 n.s.
Sum of trans FA	0.804	0.799	0.9678 n.s.

^a^Significant: *** *p* < 0.0001; ** *p* < 0.01; * *p* < 0.05; Suggestive: + *p* < 0.1; n.s.: non-significant

Despite undergoing a phase of accelerated growth, RE animals did not reach the same live weight than AL animals at slaughter age (150–160 d). This way, large differences were observed between RE and AL groups with regard to live weight, RE animals weighed about 12 kg less than AL animals at slaughter time. Such gap reduced to 10 kg of difference in carcass weights, and a suggestively (*P*-value = 0.064) lower killing out percentage was observed in RE animals (79.03% vs 80.03% in RE vs AL groups; Table 1).

As far as fatness is concerned, Duroc females fed a restricted diet tended to deposit more subcutaneous fat during the subsequent CG period than their AL counterparts (6.21 vs 5.00 mm of back fat gain at CG period in RE vs AL group; *P*-value < 0.1). However, at the age of slaughter RE gilts still showed lower back fat thickness than AL ones (22.28 mm in RE vs 25.62 mm in AL; *P*-value < 0.05) and a suggestive (*P*-value < 0.1) gain in the percentage of lean meat. Consistently with these observations, our results point out that RE animals also tend to have a lower percentage of IMF content than the AL ones (*P*-value < 0.1), at least during the CG following the restriction period.

Regarding IMF composition of the GM muscle, important differences were also observed in the muscle fatty acid profile of the two groups of animals. The most remarkable difference was the higher polyunsaturated fatty acids (PUFA) content showed by RE gilts in the CG period when compared to the AL ones (13.82% vs 11.35% of PUFA in RE vs AL pigs). Consistently, the PUFA/SFA ratio was higher in RE sows. The higher PUFA content in RE animals during CG is mainly due to an increased amount of omega-6 fatty acids in the GM of RE sows (10.83% in RE vs 8.85% in AL). The content of linoleic acid, the shortest-chained omega-6 fatty acid, and to a lesser extent the arachidonic acid content, were the main causes for these differences. Omega-3 PUFA content was also higher in the RE group, but differences did not become significant. Finally, it is worth mentioning that animals subjected to a period of food restriction tended to have a lower percentage of monounsaturated fatty acids (MUFA), including palmitoleic and oleic acids, at the end of the subsequent CG period. Conversely to IMF fatty acids profile, no differences in pH, EC nor muscle colour parameters (L*, a* and b*) were observed between RE and AL animals (data not shown).

### Transcriptome analysis of the *gluteus medius* muscle

The sequencing the 24 GM muscle samples resulted in a total of 2430 M of 75 bp paired-end reads. A general description about the total reads mapped in the sequencing process is reported in Additional file 2: Table S2. A total of 89.6% (from 88.3 to 91.6%) of reads were mapped to the porcine reference genome Sscrofa10.2, and approximately 17.4% (from 14.7 to 19.0%) of them mapped at more than one location. Of the total mapped reads, 70.6% (66.4–73.6%) corresponded to annotated genes, 80.1% (77.8–83.2%) were located in exonic regions, and 8.2% in intronic regions (7.0–9.7%). Finally, 11.7% (9.2–17.7%) of the reads were mapped in intergenic regions.

### Differentially expressed genes

**Fig. 2:**
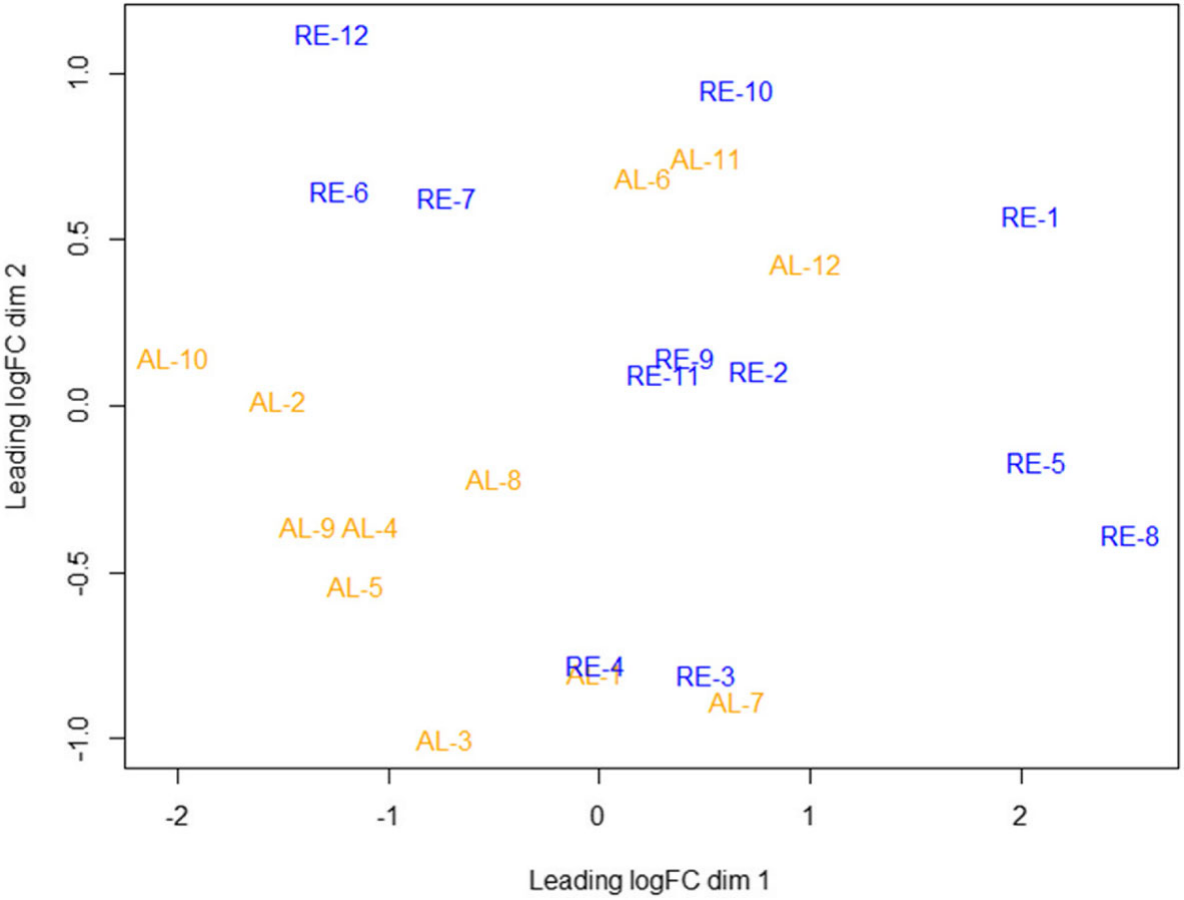
Multidimensional scaling (MDS) plot based on the log-fold-changes between each pair of RNA samples. Orange color indicates AL sows and blue color indicates RE sows

A multidimensional scaling (MDS) plot based on the log-fold-changes between each pair of RNA samples (Fig. 2) shows that biological replicates from the same group (RE and AL) still cluster together even 35 d after delivering the same nutritional treatment to RE and AL sows. This plot indicates the existence of clear differences in the muscle transcriptomic profile of these two groups of animals. However, some samples of RE and AL groups are more similar to samples of different treatments indicating that the refeeding starts to dilute the differences between both groups (Fig. 2).

Differential expression analysis using DESeq2 allowed identifying a total of 86 genes DE (ǀlog_2_FCǀ > 1, *P*_adj_-value < 0.05) between RE and AL sows (Additional file 3: Table S3). Most of these DE genes, 73 out of 86, showed higher expression levels in AL sows, whereas only 13 genes showed higher expression in RE sows. If we apply a slightly less restrictive threshold regarding FC, for example ǀlog_2_FCǀ > 0.85, the number of genes differentially expressed between groups increases to 214. However, the majority of DE genes remain to be overexpressed in AL sows: 162 genes upregulated and 52 genes down-regulated in the AL group when compared to the RE group (Additional file 3: Table S3). Prior to the functional analysis of DE genes, a detailed look of Additional file 3: Table S3 revealed that the list of the most significant DE genes contains a number of loci involved in energy metabolism and autophagy, e.g. *PFKFB3*, *MKL1*, *PPARGC1A*, *PRKAG2*, *NR4A3* or *DUSP4*.

### Functional analysis

To perform a functional classification of genes showing differential expression between RE and AL groups, a subset of 72 annotated genes having an orthologous human gene (out of the 86 genes considered as DE with FC > 2 in Additional file 3: Table S3) was submitted to IPA. The six canonical pathways most significantly (*P*-value < 0.001) enriched in the list of DE genes is shown in Table 2. These genes are involved in G-protein coupled receptor signaling, AMPK signaling, relaxin signaling, ERK/MAPK signaling, phagosome formation and type II diabetes mellitus signaling. The remaining pathways identified in the list of DE genes are shown in Additional file 4: Table S4. It is worth to highlight PPARα/RXRα activation, NRF2-mediated oxidative stress response, TR/RXR activation, IGF-1 signaling, insulin receptor signaling and triacylglycerol degradation among the canonical pathways identified.

**Table 2:** Top six canonical pathways most significantly (*P*-value< 0.001) enriched by genes differentially expressed between RE and AL groups

Canonical Pathway	-log_10_(*p*-value)	Ratio	z-score	Molecules^a^
G-Protein Coupled Receptor Signaling	4.53E00	2.57E-02	–	***SMPDL3A***, *OPRD1*, ***PDE7B***, ***PIK3R1***, ***PRKAG2***, ***DUSP4***, ***FRS2***
AMPK Signaling	4.45E00	3.17E-02	−1.633	*PFKFB3*, ***PIK3R1***, ***PRKAG2***, ***FRS2***, ***PFKFB2***, ***PPARGC1A***
Relaxin Signaling	3.85E00	3.29E-02	–	***SMPDL3A***, ***PDE7B***, ***PIK3R1***, ***PRKAG2***, ***FRS2***
ERK/MAPK Signaling	3.31E00	2.51E-02	−1.342	***PLA2G4A***, ***PIK3R1***, ***PRKAG2***, ***DUSP4***, ***FRS2***
Phagosome Formation	3.16E00	3.28E-02	–	***MRC1***, ***PIK3R1***, ***TLR1***, ***FRS2***
Type II Diabetes Mellitus Signaling	3.10E + 00	3.15E-02	−1.000	***PIK3R1***, ***CD36***, ***PRKAG2***, ***FRS2***

^a^The bold italic-lettered genes were those down-regulated in the RE group relative to the AL group

Additionally, a gene ontology analysis to identify the biological processes enriched in the set of DE genes was performed by using ClueGO. AMPK signaling and adipocytokine signalling were two of the most overrepresented KEGG pathways, and energy homeostasis was the biological process most significantly enriched in the set of DE genes. Furthermore, the regulation of lipid metabolism by PPAR alpha was also overrepresented. This way, overall results indicate that regulation of energy homeostasis plays a key role in the muscle transcriptome differences observed between RE animals and their AL counterparts during the CG process. Remarkably, the Z-score for the AMPK signaling pathway was negative (− 1.633; Table 2), indicating molecules participating in this process have the same directional effect. Indeed, almost all DE genes taking part in this pathway (*PIK3R1*, *PRKAG2*, *FRS2*, *PFKFB2,* and *PPARGC1A*) were down-regulated in the RE group compared to the AL one (Additional file 3: Table S3).

**Fig. 3:**
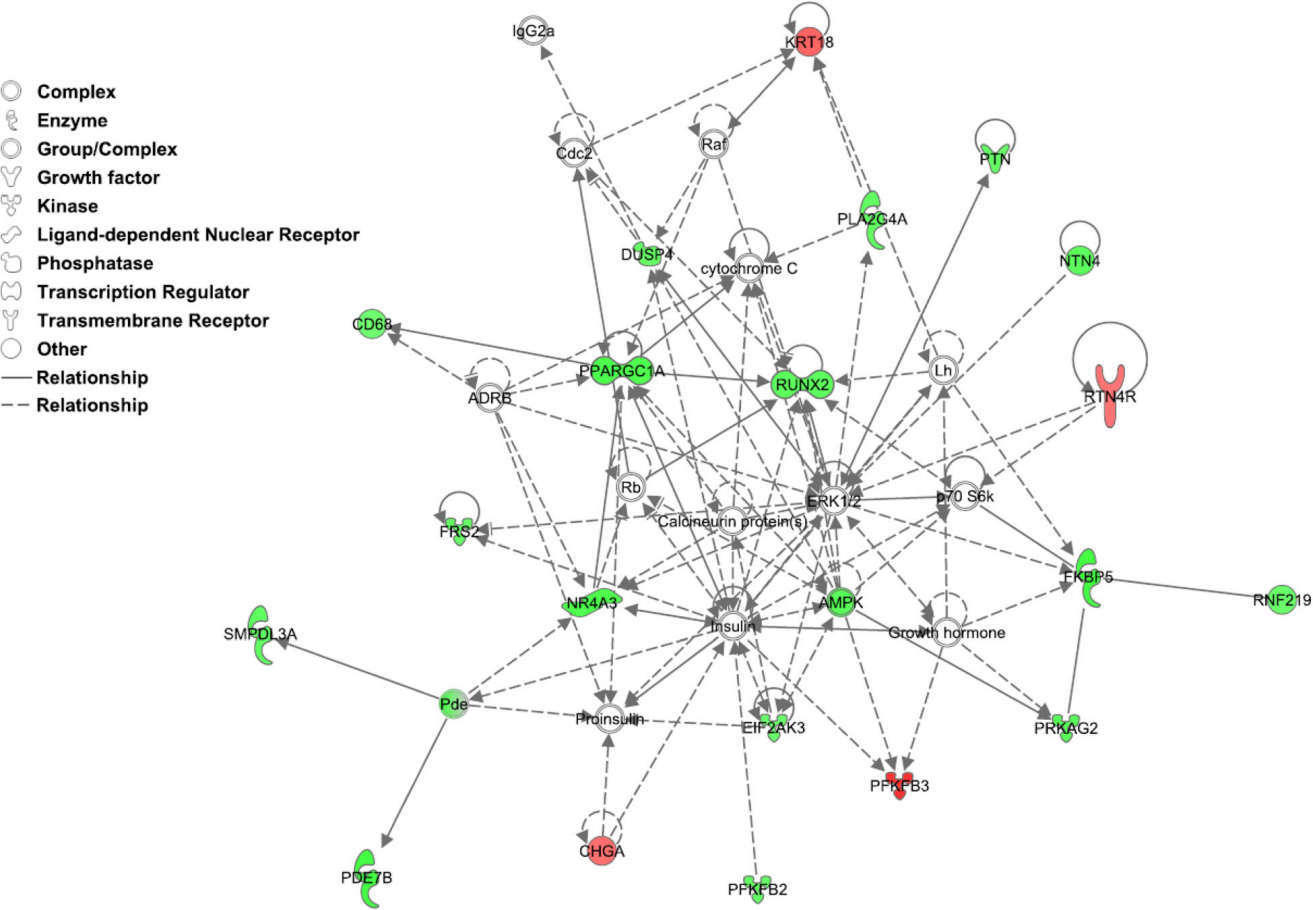
Plot of the biological network most significantly enriched by the list of genes differently expressed between animals subjected and not subjected to a feed restriction period: *Carbohydrate Metabolism, Cardiovascular System Development and Function, Cellular Development*. The shape of nodes indicates the functional classes of the gene products. The node color indicates the degree of expression: (red) up-regulated and (green) down-regulated in the RE group relative to the AL group

The most significant networks identified by IPA in the list of DE genes are listed in Table 3. Among the top five networks, three of them were associated to either carbohydrate metabolism, cardiovascular system, cell cycle and survival, or lipid metabolism, whilst the other two networks were associated to several diseases or metabolic disorders. The most significantly enriched network, mainly associated with carbohydrate metabolism, is presented in Fig. 3. Finally, among the principal functions most represented in the aforementioned identified networks (Additional file 5: Table S5), it is worth mentioning the uptake of D-glucose, glycolysis, oxidation of fatty acid, lipolysis, density of mitochondria, quantity of mitochondrial DNA, synthesis of ATP, production of reactive oxygen species, and apoptosis, among others.

**Table 3:** Top five networks enriched by genes differentially expressed between RE and AL groups. Principal functions represented in those biological networks are indicated in Additional file 5: Table S5

Associated Network functions	Score	Molecules
Carbohydrate Metabolism, Cardiovascular System Development and Function, Cellular Development	44	20
Cell Cycle, Cell Death and Survival, Glomerular Injury	28	14
Cancer, Organismal Injury and Abnormalities, Renal and Urological Disease	28	14
Lipid Metabolism, Small Molecule Biochemistry, Carbohydrate Metabolism	23	12
Auditory Disease, Hereditary Disorder, Neurological Disease	23	12

## Discussion

Despite several endocrine and metabolic studies have been conducted in several species, there is still a lack of information about the mechanisms implicated in CG in pigs. The analysis performed in the current study about the changes in the skeletal muscle transcriptome may improve our understanding about the biological and molecular mechanisms underlying CG induced by re-alimentation after a feed restriction period.

### Compensatory growth and its biological factors

In the present study, RE animals showed a CG index of 26%. This value indicates an incomplete CG, as the CG index generally ranges between 50 and 100% [13]. As previously mentioned, our experiment was designed to analyse changes in the transcriptome occurring during the CG period, and animals were slaughtered after ~35 d of re-feeding, so before completing the standard fattening and CG period to reach the commercial weight (around 120 kg of live weight in the studied Duroc population). This way, RE pigs showed lower live and carcass weights when compared to AL ones. Also viscera organ weight was 1.48 kg lower, but represented 1% more of live weight in RE vs AL pigs. In the first weeks following feed intake restriction, a relatively low basal metabolism has been observed due to the reduced weight of viscera in RE animals [13] which need some time to recover the capacity of the gastrointestinal tract [8]. Thus, during this first period of CG it is usual to observe increased live weight gains up to 20% [7].

Among the biological factors accounting for CG, changes in feed intake and feed efficiency have been reported [7, 8]. In our study, RE animals did not present an increase in feed intake after the restriction period but they showed a better feed conversion ratio during the CG phase when compared with their AL counterparts (Table 1). After feed restriction, growing pigs need about 3–4 weeks to recover the capacity and size of the gastrointestinal tract and for this reason the feed intake of those pigs increases gradually [8]. Similar results were observed in heifers when considering the whole realimentation period [22]. The better feed efficiency in RE animals might be due to a slow increase of basal metabolism as well as to lower maintenance requirements and the more efficient use of protein and energy during the beginning of realimentation [22].

Overall, we can assume that our transcriptome data offer a picture of what happens at the molecular level during the beginning of the CG period. However, we cannot discard other factors affecting the CG recovery of our animals as it has previously been described that this phenomenon varies depending on multiple factors such as the type, degree, timing and duration of growth restriction among others [7]. In the next sections, we will discuss the biological pathways and processes identified as overrepresented in the list of genes DE between RE and AL sows, thus aiming to decipher the molecular mechanisms contributing to CG and changes in muscle composition.

### Energy production and maintenance requirements

A main finding at the functional level was the down-regulation of the AMPK pathway and its downstream catabolic processes in animals that had been subjected to feed restriction (RE group). AMPK acts as a key sensor of the AMP/ATP ratio, stimulating the activation of catabolic processes to generate ATP (reviewed in [23]). In our study, the protein kinase AMP-activated non-catalytic subunit gamma 2 (*PRKAG2*) showed much higher expression levels in AL animals compared with the RE ones (FC = 2.62, *P*-value = 8.45 × 10^− 8^). The gamma subunits of AMPK are important, as they interact with AMP and ATP molecules detecting directly the cellular levels of ATP, ADP and AMP [24].

One of the immediate consequences of AMPK activation is an increase in glucose uptake and decrease in glycogen synthesis rated, coupled to an increase of glycolysis in skeletal muscle [23]. In our study, the top network identified by IPA was associated with carbohydrate metabolism (Fig. 3), whose overrepresented functions include the uptake of monosaccharide, uptake of D-glucose, metabolism of fructose-2, 6-diphosphate and glycolysis. DE genes gathered in this network were mainly up-regulated in AL group when compared with RE one (Additional file 5: Table S5). Among the set of genes under-expressed in RE animals, it is worthy to highlight the Nuclear Receptor Subfamily 4 Group A Member 3 (*NR4A3*; FC = 2.44, *P*-value = 1.03 × 10^− 5^) and 6-Phosphofructo-2-Kinase/Fructose-2, 6-Biphosphatase 2 (*PFKFB2*; FC = 2.19, *P*-value = 2.85 × 10^− 7^) genes. The *NR4A3* gene regulates the translocation of the SLC2A4 glucose transporter (also called GLUT4) to the surface of skeletal muscle cells to increase the uptake of glucose [25], while *PFKFB2* catalyses the synthesis of fructose-2, 6-bisphosphate, a key regulator of glycolysis [26].

AMPK also stimulates fatty acid oxidation and mitochondrial biogenesis to increase ATP levels through the activation of PPARG Coactivator 1 Alpha (*PPARGC1A;* also known as *PGC-1α*) in the muscle [23, 27]. *PGC-1α* is a transcriptional co-activator that, among others transcription factors, activates PPARs to induce mitochondrial gene expression and promote oxidative metabolism [28]. In our study, *PPARGC1A* was also upregulated in the AL group compared with the RE one (FC = 2.44, *P*-value = 5.52 × 10^− 5^). This agrees well with the upregulation in the AL group of genes involved in both PPARα/RXRα Activation canonical pathway and oxidation of lipid function (Additional file 3: Table S3 and Additional file 4: Table S4). In addition, we found the density of mitochondria, quantity of mitochondrial DNA, and synthesis of ATP among the overrepresented functions identified (Additional file 5: Table S5). Remarkably, while an increase of mitochondrial biogenesis to provide ATP has been described in animals under calorie restriction [29], a metabolic shift to less energy production was observed in cattle undergoing CG after restriction, with downregulation of genes involved in oxidative phosphorylation and the citric acid cycle [12]. Hence, Keogh et al. [12] indicated that mitochondrial energy production efficiency is not a relevant process contributing to the compensation of skeletal muscle tissue and overall CG in re-fed animals after restriction. This conclusion is in close agreement with the down-regulation of the AMPK pathway observed in our RE animals during the subsequent CG period. In addition, coupled with this result, we observed a decrease (z-score = − 2216) in the expression of genes involved in production of reactive oxygen species (ROS) in the RE group. Interestingly, a decrease in ROS production has also been associated to pigs showing better feed efficiency (low residual feed intake) in several studies [30, 31]. This result agrees with the improvement in the food conversion ratio of animals undergoing CG reported in our study (Table 1). Furthermore, the NRF2-mediated Oxidative Stress Response was among the canonical pathways identified by us as having DE genes downregulated in RE vs AL animals. Another mechanism to limit the production of ROS is through the autophagy degradation of mitochondria [32].

Finally, among genes upregulated in animals subjected to feed restriction, the 6-Phosphofructo-2-Kinase/Fructose-2, 6-Biphosphatase 3 gene (*PFKFB3*) resulted to be the most upregulated gene in RE vs AL animals (FC = 2.80, *P*-value = 5.26 × 10^− 7^). This gene as its paralog, the *PFKFB2* gene cited above, controls glycolysis and it has been also associated with cell proliferation and prevention of apoptosis [33]. In fact, upregulation of this gene has been described to be implicated in the Warburg effect in cancer cells to fulfil their high energy demands [34]. As a matter of fact, *PFKFB3* but not *PFKFB1, PFKFB2* nor *PFKFB4* has been identified as a novel downstream substrate of mTOR signalling pathway [35]. The mTOR pathway is essential in cell metabolism, controlling protein biosynthetic processes, growth and proliferation. This pathway, which was among the canonical pathways identified (Additional file 4: Table S4), is blocked by AMPK [23]. Interestingly, this result is in agreement with the elevated glycolytic potential observed by Gondret et al. [36] in the muscle of pigs selected for feed efficiency (low residual feed intake), in opposition to the more fatty acid oxidative profile presented by less efficient (high residual feed intake) animals. In our study, in addition to genes associated with fatty acids oxidation, also genes related with triacylglycerol degradation (*ABHD2* and *LIPG*) were identified as DE, all of them upregulated in the AL group compared with the RE one (Additional file 4: Table S4).

Altogether these results suggest that animals subjected to a feed restriction period have more glycolytic potential and less oxidative metabolic profile and catabolic processes in skeletal muscle during the subsequent CG period than animals fed ad libitum. Overall these changes may be coupled with the higher feed efficiency observed in restricted animals at the beginning of CG due to the reduced maintenance requirements.

### Skeletal muscle autophagy is downregulated during compensatory growth

Besides its role as a key sensor of the AMP/ATP ratio, the AMPK pathway plays a fundamental role in the regulation of autophagy [37], a biological process involving the degradation of cell components and molecules that are engulfed in autophagosomes that subsequently are fused with lysosomes [38]. Under stress conditions, such as dietary restriction, the recycling of macromolecules mediated by autophagy provides energy-rich compounds that can be used to restore metabolic homeostasis. From yeast to mammals, the strong activation of autophagy in response to food deprivation has been convincingly demonstrated to be a fundamental mechanism to supply nutrients to starved cells [39]. Moreover, there is evidence that autophagy plays an essential role in maintaining muscle mass and myofiber integrity [38], and that excessive activation of autophagy in the skeletal muscle (e.g. during starvation) induces a severe loss of muscular mass [40].

The downregulation of *PRKAG2*, which encodes a subunit of AMPK, in RE pigs would suggest that muscle autophagy is reduced during CG. Indeed, AMPK is a central activator of autophagy by inactivating *TORC1* and phosphorylating *ULK1* [37, 41]. In addition, we have identified six genes (*STX3*, *FKBP5*, *ACSL1*, *RUNX2*, *DUSP4* and *EIF2AK3*) that are downregulated in RE pigs (Additional file 3: Table S3) and that also activate autophagy. For instance, the Runt related transcription factor 2 (*RUNX2*) gene facilitates autophagy in metastatic cancer breast cells by increasing acetylation of α-tubulin sub-units of microtubules [42], and autophagic cell death in head and neck squamous cell carcinoma is mediated by DUSP4 (Dual Specificity Phosphatase 4) [43]. The knockdown of the Acyl-CoA Synthetase Long chain family member 1 (*ACSL1*) gene impairs cardiac autophagy [44], and the Eukaryotic Translation Initiation Factor 2 Alpha Kinase 3 (*EIF2AK3*) gene is a crucial mediator of endoplasmic reticulum stress-induced autophagy [45]. The implication of FK506 binding protein 5 (*FKBP5*) gene in promoting autophagy has been also reported [46], and Syntaxin 3 (*STX3*) gene is involved in vesicle trafficking and fusion [47].

On the other hand, in close support with our view that autophagy is decreased during CG, we have detected three genes (*KDM2B, MKL1* and *PFKFB3*) that inhibit autophagy and are upregulated in RE pigs (Additional file 3: Table S3). The downregulation of *KDM2B* (Lysine-specific demethylase 2B) in gastric cancer cells immediately induces autophagy followed by an inhibition of cell proliferation [48], and the inactivation of Megakaryoblastic Leukemia (Translocation) 1 (*MKL1*) gene in mouse embryos causes myocardial cell necrosis that could be the consequence of a decreased ability of the myocardium to cope with environmental stresses [49]. The case of the *PFKFB3* gene, the most upregulated gene in RE vs AL animals, is even more enlightening. The product of this gene controls the conversion of fructose-6-phosphate to and from fructose-2, 6-bisphosphate, a key regulator of the glycolytic enzyme phosphofructokinase-1 [50]. The inhibition of *PFKFB3* has been shown to decrease glucose uptake and to promote autophagy in cancer cells [50]. Indeed, glucose deprivation is known to activate autophagy via AMPK while mTORC1 would be concomitantly inhibited [51]. Moreover, the mTOR signalling pathway, for which *PFKFB3* has been identified as a downstream substrate, is also a key inhibitor of autophagy in response to nutritional status, growth factor and stress signals [52].

### Cellular growth and function

As stated before, *PFKFB3* has been also associated with cell proliferation and prevention of apoptosis. In the RE group, the second most upregulated gene compared with AL group was the *MKL1* gene (FC = 2.40, *P*-value = 1.10 × 10^− 8^). *MKL1* is a member of the myocardin-related transcription factor (MRTF) family which regulates a wide variety of essential biological processes such as muscle cell differentiation, cell survival and apoptosis [53]. More recently, MKL1 has been involved in the control of global transcriptional activity and cellular growth through regulating chromatin structure [54]. Although in our study genes related with anti-apoptotic function, cell proliferation and cell growth have been identified as upregulated in restricted animals, there is not an overrepresentation of cellular growth and proliferation functions in the list of genes DE between RE and AL groups.

### Fat depot and intramuscular fatty acid composition

During the restriction period, restricted gilts displayed a lower back fat gain when compared with ad libitum fed animals. In the re-alimentation period, the CG response is mainly focused to restore internal organ growth and body fat stores, although initially CG is characterized by an increased protein accretion [1, 8, 13]. In our study, RE animals also showed an increased back fat gain during CG period when compared with AL ones, but the refeeding period (~35 d) was not long enough to bridge the gap between RE and AL animals regarding fat deposition and IMF content. Similar results have been observed in pigs refed during 28 d or 34 d after restriction; therefore, it was concluded that meat eating quality was not improved in pigs under CG and that IMF content might be modulated by modifying the duration of the restriction and refeeding periods (reviewed in [1]).

Besides differences in the IMF content, also variations in the muscular fatty acids profile of sows subjected to a feed restriction period and CG were also observed in our study. While RE sows presented higher levels of PUFA content than the AL ones, and more particularly of omega-6 fatty acids (mainly linoleic acid but also arachidonic), the MUFA content, including oleic and palmitoleic acids, tended to be lower than those of AL sows. Consistently Daza et al. [2] described a higher concentration of essential fatty acids (such as linoleic acid) and a lower concentration of non-essential fatty acids in the back fat tissue of Iberian pigs subjected to severe feed restriction. These authors explained such results on the basis of a reduced activity of lipogenic enzymes. However, we did not identify lipogenic genes differentially expressed between RE and AL groups in our study. Conversely to that, RE animals presented an increase in the expression of genes related with glycolysis, cellular growth and prevention of apoptosis. We could hypothesize that our RE animals were in an initial phase of CG, characterized by the deposition of lean tissue, and therefore back fat content and IMF composition are still a reflection of the feed restriction period.

In our study, RE pigs showed an increased back fat gain in the ~35 d of re-feeding, but they were slaughtered before completing CG and reaching the commercial weight and age, so they did neither reach the point of maximum fat deposition. This circumstance prevents us from drawing conclusions about the pork composition of restricted animals in commercial fattening conditions after a longer re-feeding period.

## Conclusions

We propose that food restriction and CG constitute two contrary metabolic poles, while ad libitum feeding would represent a third intermediate condition. Our study focuses on changes in the pig skeletal muscle transcriptome profile during the phase of accelerated growth in the initial CG period after feed restriction. We have identified genes, biological processes and gene networks that may be implicated at the molecular level in the physiological mechanism of CG, and may play a role in the variations observed on IMF fatty acid profile. RE animals presented a more glycolytic potential, and less oxidative metabolic profile in muscle than AL animals. Overall these changes may be coupled with the lower food conversion ratio observed in RE animals due to the reduced maintenance requirements observed at the beginning of realimentation and CG. Our results also indicate that CG may induce a downregulation of autophagy in the skeletal muscle of pigs as a mechanism to increase muscle mass.

These results provide a new perspective about the molecular basis of CG in a monogastric species, revealing that autophagy could be an important player in pig growth. Further studies are needed to determine if AMPK pathway remains as the main mechanism underlying changes in muscle transcriptome in later phases of CG.

## Additional files

Additional file 1: **Table S1.** Composition of the fed used. (XLSX 10 kb)

Additional file 2: **Table S2.** Description of reads mapped in the RNA-Seq procedure for the 24 animals analysed. (DOCX 12 kb)

Additional file 3: **Table S3.** List of genes differentially expressed (*p*-value adjusted for multiple testing < 0.05) between animals subjected and not subjected to a feed restriction period, considering two additional thresholds regarding Fold Change (FC): a) FC > 2 (i.e. ǀlog_2_FCǀ > 1); and b) FC > 1.8 (i.e. ǀlog_2_FCǀ > 0.85). (XLSX 38 kb)

Additional file 4: **Table S4.** List of canonical pathways identified at the list of 86 genes differentially expressed (*P*_adj_-value< 0.05 and ǀlog_2_FCǀ > 1) between animals subjected and not subjected to a feed restriction period. (XLS 64 kb)

Additional file 5: **Table S5.** Principal functions represented in the biological networks identified in the list of differently expressed between animals subjected and not subjected to feed restriction. (XLS 149 kb)

## Abbreviations

a: Redness
ABHD2: Abhydrolase domain containing 2
ACSL1: Acyl-CoA synthetase long chain family member 1
ADP: Adenosine diphosphate
AL: fed ad libitum
AMP: Adenosine monophosphate
AMPK: AMP-activated protein kinase
ATP: Adenosine triphosphate
b: Yellowness
BFT: Back fat thickness
CG: Compensatory growth
DE: Differentially expressed
DNA: Deoxyribonucleic acid
DUSP4: Dual specificity phosphatase 4
EC: Electric conductivity
EIF2AK3: Eukaryotic translation initiation factor 2 alpha kinase 3
FA: Fatty acids
FC: Fold Change
FKBP5: FK506 binding protein 5
FRS2: Fibroblast growth factor receptor substrate 2
GM: *Gluteus medius* muscle
GO: Gene ontology
IGF-1: Insulin-like growth factor 1
IMF: Intramuscular fat
IPA: Ingenuity Pathway Analysis software
KDM2B: Lysine demethylase 2B
L: Lightness
LIPG: Lipase G
MDS: Multidimensional scaling
MKL1: Megakaryoblastic Leukemia (Translocation) 1 gene
mTORC1: Mammalian target of rapamycin complex 1
MUFA: Monounsaturated fatty acids
NR4A3: Nuclear receptor subfamily 4 group A member 3
NRF2: Nuclear factor, erythroid 2 like 2
PFKFB1: 6-phosphofructo-2-kinase/fructose-2, 6-biphosphatase 1
PFKFB2: 6-phosphofructo-2-kinase/fructose-2, 6-biphosphatase 2
PFKFB3: 6-phosphofructo-2-kinase/fructose-2, 6-biphosphatase 3
PFKFB4: 6-phosphofructo-2-kinase/fructose-2, 6-biphosphatase 4
pH 24: Ultimate pH
PIK3R1: Phosphoinositide-3-kinase regulatory subunit 1
PPARGC1A: PPARG coactivator 1 alpha
PPARs: Peroxisome proliferator-activated receptors
PPARα/RXRα: Peroxisome proliferator-activated receptor alpha/Retinoid X receptor alpha
PRKAG2: Protein kinase AMP-activated non-catalytic subunit gamma 2
PUFA: Polyunsaturated fatty acids
RE: fed under restriction
RNA: Ribonucleic acid
RNA-Seq: RNA sequencing
ROS: Reactive oxygen species
RUNX2: Runt related transcription factor 2
SFA: Saturated fatty acids
SLC2A4 (GLUT4): Solute carrier family 2 member 4
STX3: Syntaxin 3
TORC1: Target of rapamycin complex 1 kinase
TR/RXR: Thyroid hormone receptor/heterodimers with retinoid X receptor
ULK1: Unc-51 like autophagy activating kinase 1
w3: Omega 3
w6: Omega 6
