# Supplementary material for: Role of AMPK signalling pathway during compensatory growth in pigs
Source: BMC Genomics. 2018 Sep 17;19:682. doi: 10.1186/s12864-018-5071-5 (PMC6142327; doi:10.1186/s12864-018-5071-5)
Supplement: Supplementary file 2 — Table S2. Description of reads mapped in the RNA-Seq procedure for the 24 animals analysed. (DOCX 12 kb) [file 12864_2018_5071_MOESM2_ESM.docx]

**Table S2**. Description of reads mapped in the RNA-Seq procedure for the 24 animals analysed.

| Total number of reads (2x75pb) | 2430 M |
| --- | --- |
| Mapped reads | 89.6% (88.3%-91.6%) |
| Reads mapped in more than one location | 17.4% (14.7%-19.0%) |
| Reads mapped in annotated genes | 70.6% (66.4%-73.6%) |
| Reads mapped in exonic regions | 80.1% (74.8%-83.2%) |
| Reads mapped in intronic regions | 8.2% (7.0%-9.7%) |
| Reads mapped in intergenic regions | 11.7% (9.2%-17.7%) |
